# Sequential determination of viral load, humoral responses and phylogenetic analysis in fatal and non-fatal cases of Crimean-Congo hemorrhagic fever patients from Gujarat, India, 2019

**DOI:** 10.1371/journal.pntd.0009718

**Published:** 2021-08-30

**Authors:** Rima R. Sahay, Anita M. Shete, Pragya D. Yadav, Savita Patil, Triparna Majumdar, Rajlaxmi Jain, Dimpal A. Nyayanit, Himanshu Kaushal, Sunil J. Panjwani, Kamlesh J. Upadhyay, Chetan L. Varevadiya, Alpesh Vora, Amit Kanani, Raman R. Gangakhedkar

**Affiliations:** 1 Indian Council of Medical Research-National Institute of Virology, Maximum Containment Facility, Pune, Maharashtra, India; 2 Government Medical College and Sir-T Hospital Bhavnagar, Gujarat, India; 3 BJ Medical College and Civil Hospital, Ahmedabad, Gujarat, India; 4 Health Department, District Panchayat, Morbi, Gujarat, India; 5 Animal Husbandry Department, Foot and Mouth Disease Scheme, Ahmedabad, Gujarat, India; 6 Epidemiology and Communicable Diseases (ECD) Division, Indian Council of Medical Research, New Delhi, India; Lowell General Hospital, UNITED STATES

## Abstract

**Background:**

Thirty-four CCHF cases (17 fatal; 17 survived) were confirmed from Gujarat state, India during the year 2019. We aimed to find out the viral load, antibody kinetics, cytokine profile and phylogenetic analysis between fatal and non- fatal cases.

**Methods:**

Thirty four cases were included in this study. Blood and urine samples were collected from all the cases on the day of admission to hospital. Non-fatal cases were followed weekly for understanding the profile of viral kinetics, anti-CCHFV IgM and IgG antibodies. We also quantified the cytokines in both fatal and non-fatal cases. For epidemiological correlation, livestock were screened for anti-CCHF IgG antibodies and the tick pool specimens were tested by real time RT-PCR. Virus isolation was attempted on tick pools and human specimens and phylogenetic analysis performed on human and ticks complete genome sequences.

**Results:**

CCHF cases were detected throughout year in 2019 with the peak in August. Out of 34 cases, eight secondary CCHF cases were reported. Cases were predominantly detected in males and in 19–45 years age group (55.88%). The persistence of viremia was observed till 76^th^ POD (post onset date) in one case whereas anti-CCHFV IgM and IgG was detected amongst these cases from the 2^nd^ and 20^th^ POD respectively. Positivity observed amongst livestock and tick pools were was 21.57% and 7.4% respectively. The cytokine analysis revealed a significant increase in the level of serum IL-6, IL-10 and IFN-γ during the acute phase of the infection, but interestingly IL-10 lowered to normal upon clearance of the virus in the clinically recovered case. Fatal cases had high viral RNA copy numbers. Bleeding from one or two mucosal sites was significantly associated with fatality (OR-16.47;p-0.0034 at 95% CI). We could do CCHF virus isolation from two cases. Phylogenetic analysis revealed circulation of re-assortment of Asian-West African genotypes in humans and ticks.

**Conclusions:**

The persistence of CCHF viral RNA was detected till 76^th^ POD in one of the survivors. The circulation of a re-assortment Asian-West African genotype in a CCHF case is also reported first time from India.

## Author summary

Crimean Congo hemorrhagic fever is a zoonotic tick-borne viral hemorrhagic disease. This disease is reported from Europe, Mediterranean, north-western China, central Asia, Africa, and the Middle East. Several outbreaks of CCHF were reported from Gujarat and Rajasthan states, India from 2011 to 2019. In this study, we discuss the clinical, molecular, serological, and the cytokine data of 34 CCHF cases (17 fatal and 17 survived) which were detected from Gujarat state in the year 2019. A sequential weekly follow up of the CCHF survivors was performed to understand the viral kinetics and the antibody profile. Interestingly, the presence of persistence CCHF viral RNA was observed till 76^th^ POD in one of the survivors. To our knowledge, we are reporting this long term persistence of viremia for the first time. We also observed that the anti-CCHFV IgM detection in the serum samples starts as soon as 2^nd^ POD but anti-CCHFV IgG antibody could be detected in the majority of the cases only after the 28^th^ POD. The cytokine analysis revealed a significant increase in the level of serum IL-6, IL-10 and IFN-γ during the acute phase of the infection, but interestingly IL-10 lowered to normal upon clearance of the virus in the clinically recovered case. We did the phylogenetic analysis and concluded the circulation of the Asian-West African re-assortment genotype in humans, which has not been reported from India prior to this study.

## Introduction

Crimean congo hemorrhagic fever (CCHF) is a tick-borne zoonotic disease belonging to the family *Nairoviridae*, transmitted by tick bite, or through contact of body fluid of infected livestock and humans [1]. CCHF causes severe viral hemorrhagic fever outbreaks and the case fatality rate (CFR) ranges from 3 to 60% [2]. This disease is reported from Europe, Mediterranean, north-western China, central Asia, Africa, and the Middle East [1]. The majority of cases have been reported amongst people involved in livestock-related work, such as agricultural/farming, grazing animals, milking, slaughtering and veterinarians [2]. Recently the incidences of CCHF have been increasing rapidly and many sporadic human cases and focal outbreaks are reported from the different countries [2].

In India, CCHF cases were confirmed during the period, 2011 to 2019 from Gujarat state and in 2014, 2015 and 2019 from Rajasthan state [3,4,5]. The clinical presentation starts with fever, myalgia, diarrhoea and vomiting during the prodromal phase, progresses to ecchymoses, petechial rash, bleeding from mucosal/punctured sites, coagulation disorders, fatal hemorrhagic manifestations including disseminated intravascular coagulation and death [6,7,8,9]. Direct or indirect damages occur to the endothelial cells either by virus or of chemokines/cytokines release [7]. CCHF has an overlapping clinical profile with dengue hemorrhagic fever making it very difficult to diagnose in the early stages. Because of the severity, lack of specific prophylactic/therapeutic options and its epidemic potential, CCHF was included in World Health Organization priority list of diseases needing urgent research and development attention [10].

Serum pro-and anti-inflammatory cytokines like Tumor Necrosis Factor (TNF)-α, Interleukin (IL)-6, IL-10, IL-12, and (Interferon) IFN-γ were elevated in CCHF patients and levels were higher in the fatal cases, suggestive of their prominent role in the fulminant disease progression [11,12,13,14,15]. The data presented in these studies included analysis of serologic, virologic and cytokines (IL-6, IL-10, TNF-α, and IFN-γ) as per the availability of the samples at three set points till 7^th^ day [14]. They reported the mean serum CCHFV titer at admission was 5.5E + 09 copies/mL in the fatal cases and 5.7E + 08 copies/mL in the survivors [14].

In the current study we did systematic follow-up of CCHF survivors and compared our finding with the fatal cases to generate clear evidence on the viral RNA kinetics, antibody response and the cytokine profiles. Phylogenetic analysis was performed using Next Generation Sequencing (NGS) to understand the circulation of the CCHFV strains in humans and ticks. We also tried to explore the variation in amino-acid sequences between fatal and survived cases.

## Material and methods

### Ethical statement

Written and informed consent was obtained prior to collection of clinical data and samples from suspected and confirmed CCHF cases and from relatives, if the patients were unable to consent. For children, the written consents were obtained from their parent/guardian. The study was approved by the Institutional Human Ethics Committee at the ICMR-NIV, Pune (IHEC Number-NIV/IHEC/2018/March/D-2).

### Identification of suspected CCHFV cases

In 2019, a total of 124 suspected CCHF cases were identified, those fulfilling the case definition as described by national CCHF guidelines [9]. The clinical information was gathered using the dedicated VHF clinical sheet. For this study, a CCHF primary case was defined as the occurrence of the first laboratory confirmed case in the particular community/area. A CCHF secondary case was defined as the occurrence of a laboratory confirmed CCHF case due to close contact within 14 days after onset of illness in the primary case patient. A cluster of CCHF cases was defined as the aggregation of laboratory confirmed cases in a defined subpopulation.

### Collection of samples from Humans, livestock and tick pools

Indian Council of Medical Research-National Institute of Virology (ICMR-NIV), Pune, India is a referral laboratory for the diagnosis of CCHF. Blood in ethylenediaminetetraacetic acid (EDTA) medium, serum and urine of 124 suspected CCHF cases were referred from tertiary care hospitals of Gujarat state to ICMR-NIV for the diagnosis. The dengue, chikungunya and zika virus infections were ruled out using CDC trioplex real time RTPCR assays. A prospective follow-up study was performed for CCHF survivors (n = 17), till viremia was detected in the blood and then patients were discharged from the hospital, which included weekly clinical status, screening of blood and urine samples for viral RNA and serum samples for anti-CCHFV IgM and IgG antibodies. Each patient had presented at different post-onset day (POD) [i.e, from date of onset of symptoms to date of sample collection] and hence the follow up time point for each is different as described in the S1 Table. Semen samples of two cases (cases number 32 and 34) were also collected on POD 32^nd^ and 55^th^.

Livestock samples (cattle, goat, sheep, bull) (n = 241) and *Hyalomma annaloticum* tick-pools (n = 162) from the household of confirmed CCHF cases were collected under the surveillance programme of Foot and Mouth Disease Scheme, Animal Husbandry Department, Ahmedabad, Gujarat state, India and tested for CCHFV.

### Testing of CCHF viral RNA by real-time reverse transcriptase-polymerase chain reaction (qRTPCR) and anti-CCHFV IgM and IgG antibodies by Enzyme-linked Immunosorbent Assay (ELISA)

RNA was extracted from the EDTA blood, urine, semen and tick-pool (*Hyalomma annaloticum*) homogenates using the Magmax RNA extraction kit (Applied Biosystems, USA) as per manufacturer’s instructions and were tested by CCHFV qRTPCR [16]. Serum samples of CCHF cases were tested for anti-CCHF IgM and IgG antibodies by ELISA while livestock [cow, buffalo, goat, sheep, bull, calf] were tested for anti-CCHF IgG antibodies by ELISA [3,17]. All the infectious work was performed in containment facility of ICMR-NIV, Pune.

### Quantification of cytokine level in serum samples of CCHFV cases

The serum samples of the acute phase of fatal and survivors upon recovery (at lowest viremia phase) were examined for the cytokine profile. Acute phase of fatal cases (AFC) were defined as cases with acute onset illness (POD ≤ 10 days) whose outcome was fatal. Acute phase of survived cases (ASC) were defined as cases with acute onset illness (POD ≤ 10 days) who eventually survived. The level of cytokines IL-2, IL-4, IL-6, IL-10, IFN-γ, TNF-α and IL-17A were analyzed using BD Cytometric Bead Array (CBA) Human Th1/Th2/Th17 Cytokine Kit, as per manufacturer’s instructions. Cytokine levels were measured on a BD FACS Calibur flow cytometry (BD Biosciences) using BD CellQuest Pro software. The data were analyzed using flow Cytometric analysis program (FCAP) Array software. The detection sensitivity of IL-2, IL-4, IL-6, IL-10, IFN-γ, TNF-α and IL-17A were 2.6, 4.9, 2.4, 4.5, 3.8, 3.7 and 18.9 pg/ml respectively.

### Statistical analysis

The clinical data (symptoms, signs and laboratory parameters) as well as epidemiological data (month, district, history of tick bite, livestock contact, primary or secondary case, travel history) were collected. Along with this, demographic data (age, sex, occupation, relationship with primary/index cases) were also collected. Outcomes measured were different clinical presentation in fatal and survived cases. To understand the exposure details about tick bite, livestock contact and occupation were collected from each CCHF cases. Different demographic variables like age and sex were also collected to understand any specific predominance. The viral load, antibody kinetics were also collected and results were compared with fatal cases and survivors. Figures related to viral copy numbers, optical density (OD) of anti-CCHFV IgM and IgG were generated using GraphPad Prism software version 8.4.3 (GraphPad, San Diego, California). We used Epi-Info version 7.2 for calculation of proportions, p values, and Odds Ratio (OR) at 95% confidence intervals (CI). For cytokine data, we tested the null hypothesis that there was no difference in the serum cytokine level at the acute phase of CCHF and upon recovery. In the present study, we had four different study groups which were unpaired. Since, the cytokine data was not following normal distribution and the study groups were independent, we used non-parametric Kruskal–Wallis test followed by the post-hoc Dunn’s multiple comparison test to analyses the data.

### NGS and phylogenetic analysis

For next generation sequencing of clinical specimens input RNA was quantified using Qubit 2.0 Fluorometer (Thermo-Fisher, Grand Island, NY, USA) followed by library preparations and quantification [18]. The quantified libraries were loaded to the NGS platform (Illumina, USA). The reads generated were analyzed using CLC Genomics Workbench version 20.0.4 (CLC, Qiagen). Reference-based mapping was performed.

Phylogenetic tree generated using MEGA Software (version X) sequences retrieved from this study and the CCHFV genomic segments downloaded from the Genbank (n = 38). A maximum-likelihood tree was generated for all three segments of CCHFV using the General time reversible model. A bootstrap replication of 1000 cycles was performed to assess the statistical robustness of the analysis. The percentage of nucleotide and amino acid differences for each gene was calculated with respect to its reference gene sequence.

### Virus isolation from human and tick pools

One hundred microliters of serum samples of twenty-four cases with viral RNA copy number > 5.1 x 10^4^ as well as twelve CCHFV positive tick-pools were inoculated onto 24-well *Vero CCL-81* cell monolayers maintained in MEM (Gibco, UK) and observed for one week [1] and confirmed by qRTPCR in two continuous passages [15].

## Results

### Clinical presentation

Incubation period for CCHF infection was recorded as 2–12 days since the (mean 6.14 days ± 2.59 SD). Cases were observed more amongst male (61.76%) and between the age group of 19–45 years (55.88%) (Table 1). Out of 34 cases reported, 17 cases succumbed to the disease (CFR 50%). The earliest median day of admission of the fatal CCHFV cases was 6.4 days and non-fatal cases was 5.8 days. But there was no statistical significance of outcomes (recovery vs fatality) with respect days from the onset of symptoms to hospital admission. All the fatalities happened during hospital admission within 1–3 days. The survivors showed clinical resolution from the day 7–14 post-admission (mean 9.77 days ± 2.11 SD). All the cases presented with mild to high-grade fever (ranging from 99^0^ to 103^0^ F). 76.4% had mild, 17.64% had moderate while 5.88% had high-grade fever. No statistical significance was observed in grades of fever with fatality. Other symptoms and signs included vomiting (35.29%), myalgia (29.41%), headache, (20.58%), diarrhea (14.70%), anorexia (14.70%) and altered sensorium (14.70%) (Table 2). Bleeding was observed in 24 cases (16 fatal and 8 survived) suggestive of damage to the endothelial cells. Bleeding was noted most commonly from one or two mucosal sites predominantly from the gums (41.66%) and hematochezia (20.83%) (Table 2). Bleeding was significantly associated with fatality; OR 16.47 (p values 0.0034 at 95%CI). Similarly, fatal CCHF cases had reported bleeding gums which was found significantly associated with mortality (p value of 0.05483 at 95%CI and OR of 8.23). No other co-morbid conditions were reported from the patients, except Diabetes mellitus type-II (controlled) in two cases.

**Table 1:** Details of CCHF positive cases (n = 34), clusters of cases, clinical outcome, along with livestock and tick pool positivity from different districts of Gujarat state.

Cases Number.	Month	Clustering of cases	Districts from Gujarat	Age (in years)	Sex	Post onset date (POD) (earliest date of admission)	Cyclic threshold (Ct) value	Viral RNA copy numbers per ml	Anti-CCHFV IgM	Sum of optical density (OD) at 450 nanometer	Outcome	Occupation	Tick bite history	Livestock contact	Anti-CCHFV IgG Livestock Positive/Tested	CCHFV real time-RT-PCR Tick pool positive/Tested	Primary/Secondary cases	Laboratory Identity Number	L gene Accession Number	M gene Accession Number	S gene Accession Number
1	Feb	..	Rajkot	33	M	6	22	8.9 x 10^6^	+	0.52	D	Farmer	Nk	Y	0/0	0/0	Primary	MCL-19-H-99	MN866129	MN866130	MN866131
2	Mar	..	Bhavnagar	48	M	8	25	1.2 x 10^6^	+	0.33	D	Farmer	Y	Y	0/24	0/27	Primary	MCL-19-H-241	MN866132	MN866133	MN866134
3	Mar	..	Bhavnagar	50	M	6	25	1.2 x 10^6^	-	0.12	S	Farmer	Y	Y	0/11	0/11	Primary	MCL-19-H-387	MN866150	MN866151	MN866152
4	May	..	Botad	70	F	9	27	3.5 x 10^5^	+	0.87	D	Homemaker (cattle handler)	Nk	Y	0/2	04-Sep	Primary	MCL-19-H-924	MN866135	MN866136	MN866137
5*	July	..	Bhavnagar	48	M	6	20	3.2 x 10^7^	+	0.38	D	Farmer	Nk	Y	0/0	0/0	Primary	MCL-19-H-1565	MN866138	MN866139	MN866140
6	July	..	Bhavnagar	28	M	6	25	1.2 x 10^6^	+	0.31	S	Farmer	Y	Y	0/5	0/5	Primary	MCL-19-H-1574	MN866153	MN866154	MN866155
7	July	..	Bhavnagar	50	F	2	18	1.1 x 10^8^	+	0.43	D	Homemaker (cattle handler)	Nk	Y	0/0	0/0	Primary	MCL-19-H-1710	MN866141	MN866142	MN866143
8*	Aug	..	Surendranagar	75	F	7	17	2.2 x 10^8^	+	0.26	D	Homemaker (cattle handler)	Nk	Y	0/3	0/3	Primary	MCL-19-H-1782	NR	NR	NR
9	Aug	Cluster-1	Bhavnagar	35	F	5	19	6.2 x 10^6^	+	0.23	D	Homemaker	Nk	Y	05-Jun	0/6	Primary	MCL-19-H-1789	MN866144	MN866145	MN866146
												(cattle handler)									
10	Aug	Cluster-1	Bhavnagar	40	F	2	23	4.6 x 10^6^	-	0.09	S	Homemaker	Nk	Y	..	..	Secondary	MCL-19-H-1882	MN866159	MN866160	MN866161
												(cattle handler)									
11	Aug	Cluster-2	Morbi	20	M	5	..	..	+	0.613	D	Factory Worker	Nk	Nk	0/8	0/1	Secondary	MCL-19-H-1796	NR	NR	NR
12	Aug	Cluster-2	Morbi	19	M	4	..	..	+	0.57	S	Factory Worker	Nk	Nk	..	..	Secondary	MCL-19-H-1798	NR	NR	NR
13	Aug	Cluster-3	Surendranagar	45	F	6	23	4.6 x 10^6^	+	0.59	D	Homemaker	Nk	Y	02-Jun	0/6	Primary	MCL-19-H-1812	MN866147	MN866148	MN866149
												(cattle handler)									
14	Aug	Cluster-3	Surendranagar	95	F	3	26	6.7 x 10^5^	+	0.24	S	Homemaker	Nk	Y	..	..	Secondary	MCL-19-H-1816	MN866156	MN866157	MN866158
												(cattle handler)									
15	Aug	Cluster- 4	Jamnagar	27	F	4	29	9.7 x 10^4^	-	0.184	S	Doctor	No	No	0/12	0/1	Secondary	MCL-19-H-1920	MN866165	MN866166	MN866167
16	Aug	Cluster- 4	Jamnagar	33	M	11	34	3.8 x 10^3^	+	1.15	S	Medical store keeper	Nk	Nk	..	..	Secondary	MCL-19-H-2028	MN866174	MN866175	MN866176
17	Aug	..	Botad	47	F	9	20	3.2 x 10^7^	+	0.38	D	Homemaker	Nk	Y	Mar-13	0/3	Primary	MCL-19-H-2006	MN866171	MN866172	MN866173
												(cattle handler)									
18^**#**^	Sep	..	Rajkot	42	M	7	30	5.1 x 10^4^	-	0.18	D	Farmer	Y	Y	03-Jun	0/2	Primary	MCL-19-H-2076	MN866177	MN866178	MN866179
19^**#**^	Sep	..	Kheda	55	M	11	22	8.9 x 10^6^	+	0.34	D	Farmer	Nk	Y	0/32	0/2	Primary	MCL-19-H-2524	MN866183	MN866184	MN866185
20	Sep	..	Bhavnagar	42	M	6	22	8.9 x 10^6^	-	0.132	S	Farmer	Nk	Y	May-20	Feb-16	Primary	MCL-19-H-2757	NR	NR	NR
21	Sep	..	Amreli	65	F	5	23	4.6 x 10^6^	+	0.88	D	Homemaker	Nk	Y	Feb-20	0/20	Primary	MCL-19-H-2856	MN930405	MN930416	MN930426
												(cattle handler)									
22^**#**^	Sep	Cluster- 5	Bhavnagar	38	M	8	33	7.3 x 10^3^	+	1.104	S	Farmer	Nk	Y	03-May	01-May	Secondary	MCL-19-H-2928	NR	NR	NR
23	Oct	Cluster- 5	Bhavnagar	29	F	2	31	2.6 x 10^4^	+	0.745	S	Homemaker	Nk	Y	0/5	0/5	Primary	MCL-19-H-3068	MN930403	MN930418	MN930428
												(cattle handler)									
24	Oct	Cluster- 5	Bhavnagar	13	F	8	36	1.0 x 10^3^	+	1.37	S	Student	No	No	..	..	Secondary	MCL-19-H-3081	MN930402	MN930419	MN930429
25	Oct	..	Bhavnagar	29	M	8	36	1.0 x 10^3^	+	1.17	S	Panshop owner	Nk	Y	..	..	Primary	MCL-19-H-3078			
26	Oct	..	Surendranagar	43	M	9	30	5.1 x 10^4^	+	0.78	S	Herdsman	Nk	Y	06-Jun	0/3	Primary	MCL-19-H-3075	MN930404	MN930417	MN930427
27	Oct	..	Bhavnagar	53	M	7	25	1.2 x 10^6^	+	1.27	D	Farmer	Nk	Y	06-Jul	0/1	Primary	MCL-19-H-3092	MN930408	MN930413	MN930423
28	Oct	..	Amreli	60	M	12	35	2.0 x 10^3^	+	1.314	S	Cattle rarer	Nk	Y	10-Oct	02-May	Primary	MCL-19-H-3108	NR	NR	NR
29^**#**^	Oct	..	Bhavnagar	17	M	4	20	3.2 x 10^7^	-	0.15	S	Student	Nk	Y	01-May	02-May	Primary	MCL-19-H-3110	MW298534	MW298540	MW298548
30	Nov	..	Bhavnagar	40	F	4	25.21	1.1 x 10^6^	+	0.22	D	Homemaker	Y	Y	02-Jun	0/6	Primary	MCL-19-H-3154	MW298535	MW298542	MW298549
												(cattle handler)									
31	Nov	..	Rajkot	65	M	7	27.34	2.8 x 10^5^	+	0.924	D	Farmer	Nk	Y	0/5	0/1	Primary	MCL-19-H-3173	MW298539	MW298543	MW298550
32^**#**^^**%**^	Nov	..	Amreli	40	M	4	28	1.8 x 10^5^	+	1.42	S	Farmer	Nk	Y	0/10	0/10	Primary	MCL-19-H-3232	MW298536	MW298544	MW298551
33	Nov	..	Anand	20	M	5	20.25	2.7 x 10^7^	-	0.2	D	Student	Nk	Nk	0/6	01-Jan	Primary	MCL-19-H-3460	MW298537	MW298545	MW298552
34%	Dec	..	Bhavnagar	24	M	3	28.71	1.1 x 10^5^	+	1.267	S	Farmer	Y	Y	04-Aug	0/8	Primary	MCL-19-H-3508	MW298538	NR	MW298553

(+) Positive; (-) Negative; (M)- Male; (F)- Female; (D)- Death; (S)- Survived; (Y)- Yes; (N)-No; (Nk)- Not known

Cluster-1-Case-10 secondary case from the primary case-9

Cluster-2-Cases-11 and 12 were the secondary cases from the suspected CCHF death case in the same factory.

Cluster-3-Case-14 was the secondary case from the primary case-13

Cluster-4-Case-15 and case-16 were the secondary case from the suspected CCHF death case.

Cluster-5-Case-22 and Case-24 were secondary cases from the primary case-23

Livestock samples of Cow, buffalo, goat, sheep, bull, calf were tested for Anti-CCHFV IgG by ELISA

^#^ Urine samples of the cases 18, 19, 22, 29 and 32 were positive for CCHFV RNA by qRTPCR assay with Ct value (viral RNA copy number/ml) of 30 (5.1 x10^4^), 35 (2.0 x 10^3^), 34.5 (2.8 x10^3^), 36 (1.0 x 10^3^), 37 (5.5 x 10^2^) respectively on first collection. All follow up urine samples of the survival cases were—for CCHFV RNA by qRTPCR.

^%^ Semen samples of cases-32 and 34 were found—for CCHFV RNA by qRTPCR on 32^nd^ and 55^th^ POD

*Successful virus isolation from the serum samples of the case numbers- 5 and 8

All clinical samples of CCHF positive cases were tested and found to be—for anti-CCHFV IgG antibodies at first collection time point.

Sum OD should be <0.2 for IgM antibody positive for CCHF case

(..) No data; NR- Sequence for the segment could not be retrieved

**Table 2:** Clinical presentation of fatal and survived CCHFV cases.

CCHF Cases	CCHFV cases (n = 34) (%)	Fatal cases (n = 17)	Survived cases (n = 17)	Odds Ratio (95% Confidence Interval)	p value (2 tailed)
**Signs and Symptoms**					
Fever	34/34 (100)	17/17	17/17	..	..
Headache	7/34 (20.58)	0/17	7/17	NC	NC
Myalgia	10/34 (29.41)	6/17	4/17	1.74 (0.3781–8.693)	0.4834
Arthralgia	3/34 (8.82)	1/17	2/17	0.48 (0.01502–6.855)	0.6136
Abdominal pain	3/34 (8.82)	1/17	2/17	0.48 (0.01502–6.855)	0.6136
Nausea and vomiting	12/34 (35.29)	5/17	7/17	0.60 (0.135–2.574)	0.5012
Diarrhoea	5/34 (14.70)	1/17	4/17	0.21 (0.007–1.925)	0.1899
Anorexia	5/34 (14.70)	1/17	4/17	0.21 (0.007–1.925)	0.1899
Fatigue	2/34 (5.88)	0/17	2/17	NC	NC
Breathlessness	2/34 (5.88)	2/17	0/17	NC	NC
Altered sensorium	5/34 (14.70)	3/17	2/17	1.59 (0.207–15.02)	0.67
Jaundice	2/34 (5.88)	2/17	0/17	NC	NC
Bleeding tendencies*	24/34 (70.58)	16/17	8/17	16.47 (2.185–419.7)	0.00345
• Haemoptysis	1/24 (4.16)	0/16	1/8	NC	NC
• Haematuria	1/24 (4.16)	0/16	1/8	NC	NC
• Hematemesis	4/24 (16.66)	2/16	2/8	0.45 (0.0384–5.125)	0.4980
• Bleeding gums*	10/24 (4.16)	9/16	1/8	8.23 (0.9639–225)	0.05483
• Epistaxis	1/24 (4.16)	0/16	1/8	NC	NC
• Bleeding per vaginum	1/24 (4.16)	1/16	0/8	NC	NC
• Haematochezia	5/24 (20.83)	2/16	3/8	0.26 (0.0241–2.177)	0.2134
• Bleeding at injection site	1/24 (4.16)	1/16	0/8	NC	NC
• Ecchymoses	4/24 (16.66)	3/16	1/8	1.59 (0.1401–48.27)	0.7642
• Petechial rash	1/24 (4.16)	1/16	0/8	NC	NC

NC- Cannot be calculated as one of the cells has zero value

*Clinical presentation of bleeding was found to be significantly associated with fatality (p value of 0.00345 at 95% CI and OR of 16.47)

*Similarly, fatal CCHF cases had reported bleeding gums which was found significantly associated with mortality (p value of 0.05483 at 95% CI and OR of 8.23)

Laboratory findings revealed leucopenia (1200/ μL- 4000/ μL) in twenty cases and severe thrombocytopenia (7000/ μL-95000/ μL) with underlying bleeding disorder in all the cases (S2 Table). The level of serum aspartate aminotransferase (AST) and alanine aminotransferase (ALT) were elevated in all the cases (S2 Table). Prothrombin time (PT), International Normalized Ratio (INR) and activated partial thromboplastin time (aPTT) were elevated in all the twenty-four cases with bleeding, suggestive of deranged coagulation parameters (S2 Table). Blood urea and serum creatinine were elevated only in three fatal cases (cases numbers-1, 18, 19) and one survived case number-32, suggestive of acute kidney injury (S2 Table). All the patients were started on ribavirin therapy immediately as per the national CCHF management guidelines. Along with that the supportive treatment was provided which focused on maintaining the fluid and electrolyte balance and the ventilator support for maintaining the oxygen saturation. Depending on the clinical presentation, inotropic drugs were given along with the transfusion of the packed cell volume/fresh frozen plasma for providing the hemodynamic support. Broad spectrum antibiotic therapy were also initiated to prevent/treat any secondary infections.

### Cluster of CCHF cases

**Fig 1:**
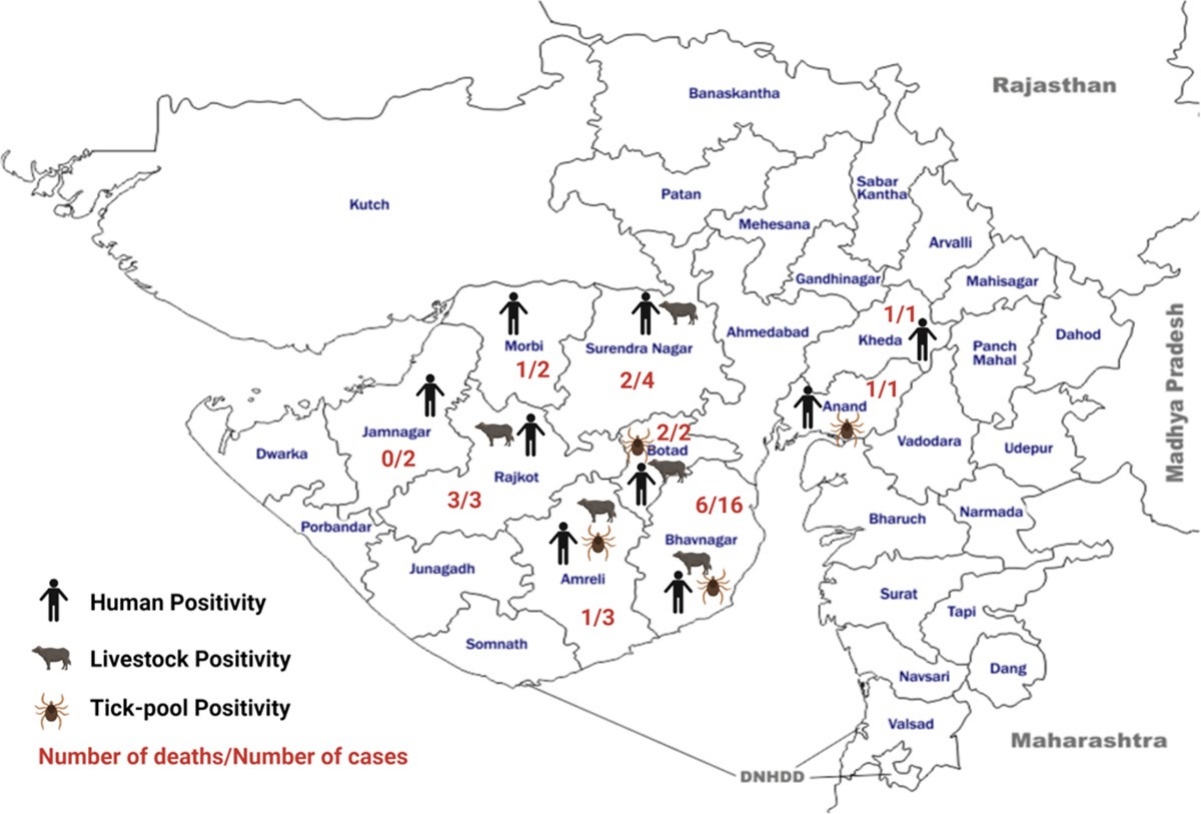
District-wise CCHF positivity in the humans, tick-pools and livestock from Gujarat State, India during the year 2019. Image attribution: https://d-maps.com/carte.php?num_car=8623&lang=en. License attribution: https://d-maps.com/conditions.php?lang=en. The map was modified showing the district-wise CCHF human cases, tick-pools and livestock positivity in the year 2019. All the details provided in the figure are created by the author themselves using the licensed version of the online software Biorender.com.

**Fig 2:**
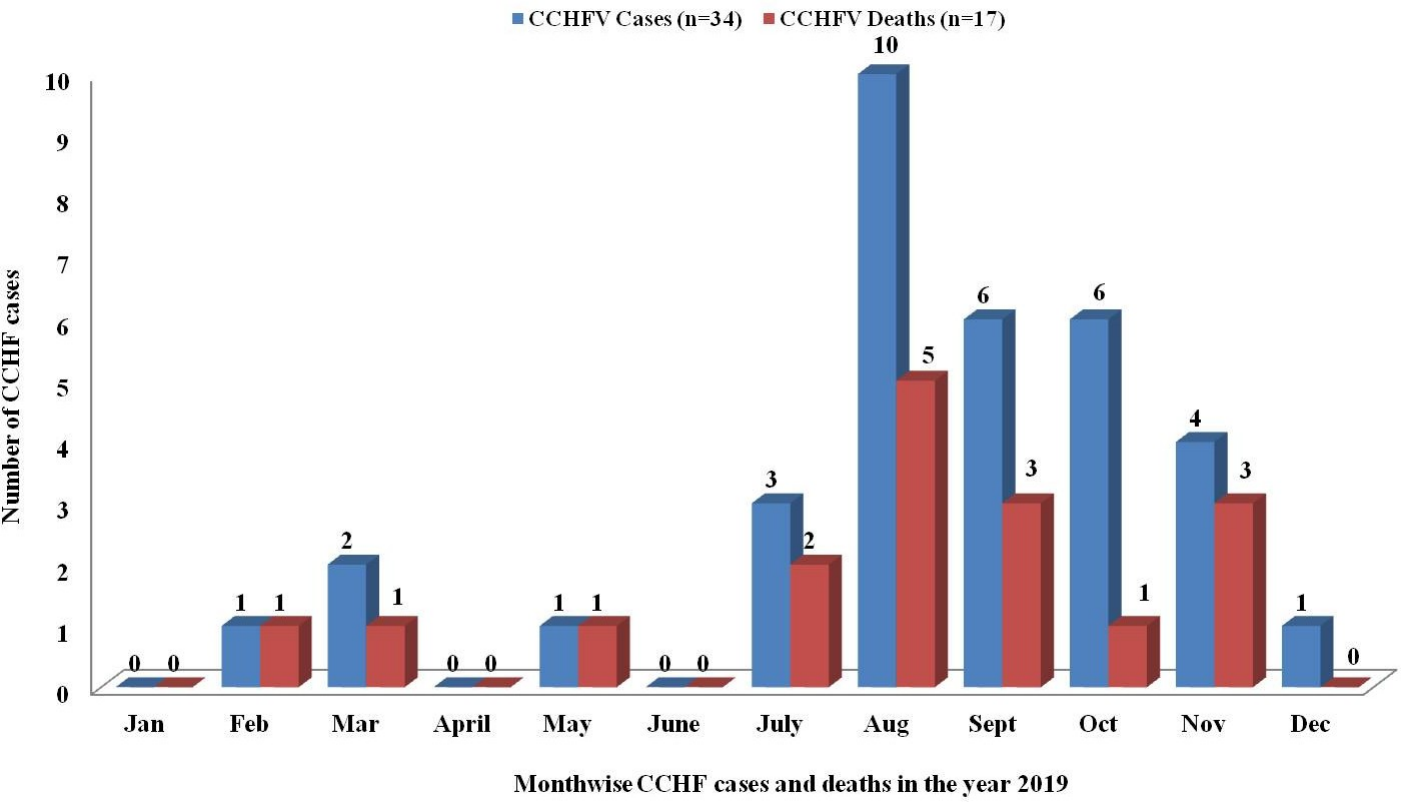
Month-wise distribution of CCHF cases and deaths in Gujarat state during 2019.

Thirty-four CCHF cases were reported from nine districts (Bhavnagar, Surendranagar, Rajkot, Morbi, Jamnagar, Amreli, Anand, Kheda and Botad) of Gujarat state in 2019 (Fig 1). Sporadic cases of CCHF were detected throughout the year with an increase in the incidence of cases in August followed by September and October months (Fig 2). Twenty-eight out of 34 cases (82.35%) had a history of livestock contact but only six cases (case numbers-2, 3, 6, 18, 30 and 34) recalled and gave definitive tick bite history in preceding ten days before the onset of symptoms. No history of recent travel in the preceding three months in any cases. Clustering of cases amongst the close contacts and health care workers were also observed from Morbi, Surendranagar, Jamnagar and Bhavnagar districts (Table 1). Out of 34 cases, 8 secondary cases were reported with possibility of human to human transmission by exposure to body fluids including vomitus and blood of infected cases (Table 1). Five clusters of human to human transmission were observed. All secondary cases with human to human transmission had less viremia and all had survived.

#### Cluster-1 [Case-10 as secondary case from the primary case-9]

40 year female, homemaker from Bhavnagar district, close contact case-9, was exposed during the patient care activities. *Cluster-2 [Cases-11 and 12 were the secondary cases from the suspected CCHF death case in the same factory]*: In Morbi district, two factory workers tested positive for anti-CCHF IgM antibodies. When the history was elicited, there were no definitive tick bites or animal contacts. They reported to had contact with a suspected CCHF case (co-worker) who died a week back before they developed symptoms (exposed during patient care and during cremation).

#### Cluster-3 [Case-14 was the secondary case from the primary case-13]

In Surendranagar district, 95 year old female tested positive for CCHF. When detailed history was elicited, she gave contact with the suspected CCHF death case (her daughter in law) 5 days before onset of her own symptoms. The retrospective samples of the case-14 were tested and were found positive for CCHF (exposed during patient care and during last ritual activities).

#### Cluster-4 [Case-15 and case-16 were the secondary case from the suspected CCHF death case]

One health care worker from tertiary care hospital (examined the suspected CCHF death case) and one close relative (brother of the same suspected CCHF death case) from Jamnagar district tested positive for CCHF (exposed to vomitus and blood while examining and patient care activities).

#### Cluster-5 [Case-22 and Case-24 were secondary cases from the primary case-23]

Two family members including niece (case-24) and husband (case-22) developed symptoms 3 to 4 days of contact with CCHF case-23 (exposed to the body fluids including blood, vomitus and stool while performing patient care activities).

### Viral load in the blood, urine and semen of the CCHF cases

**Fig 3:**
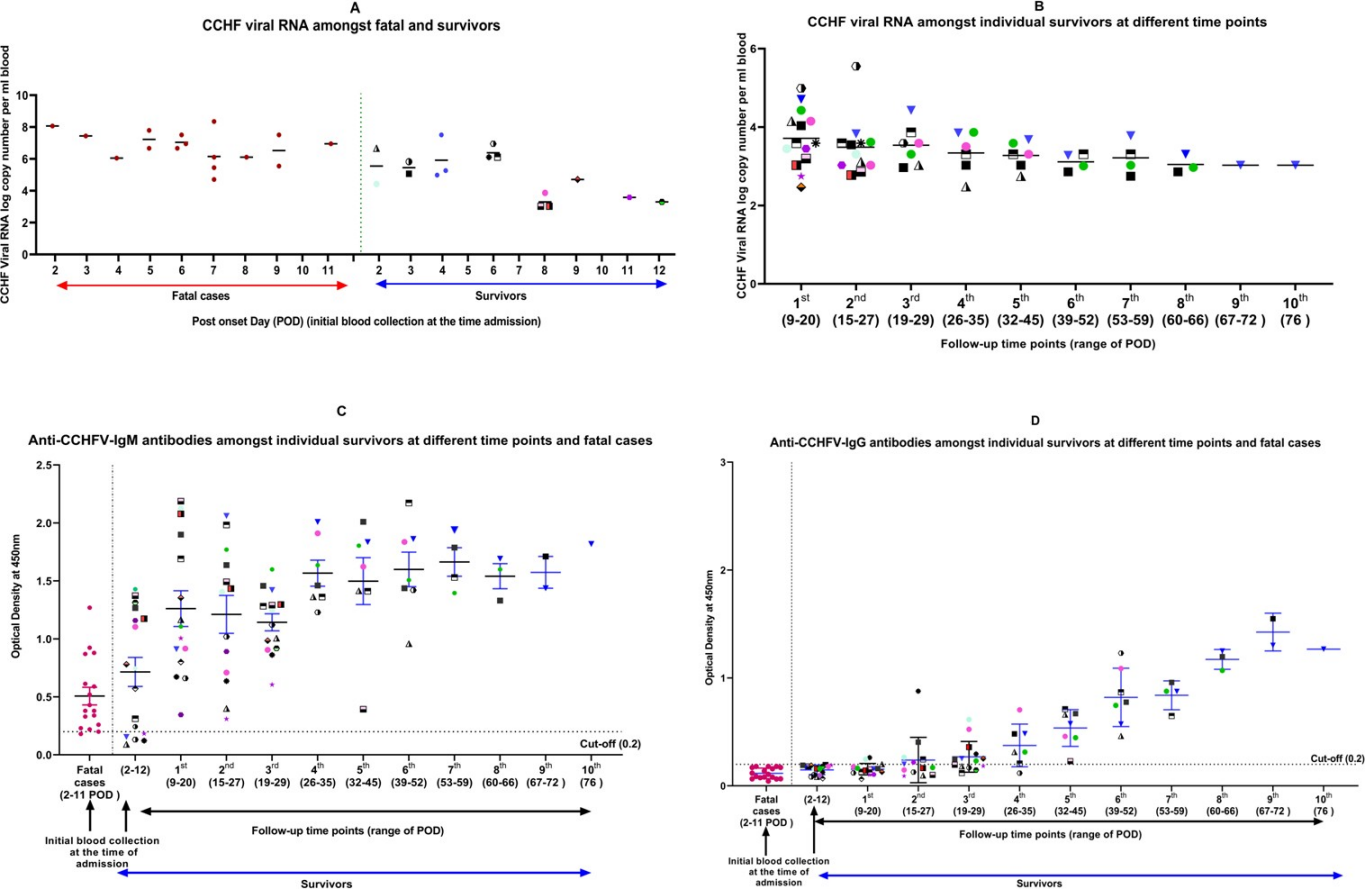
CCHF Viral RNA, anti-CCHFV IgM and anti-CCHFV IgG kinetics of CCHFV cases. (A) CCHF viral RNA amongst fatal and survivors (B) CCHF viral RNA amongst individual survivors at different time points (C) Anti-CCHFV-IgM antibodies amongst individual survivors at different time points and fatal cases (D) Anti-CCHFV-IgG antibodies amongst individual survivors at different time points and fatal cases.

Out of 124 suspected cases 34 (17 fatal; 17 survivors) were found positive for CCHFV. The mean viral RNA copy number in fatal cases was 7.2 x 10^6^ while in survivors was 1.0 x10^5^ (Table 1). Viral load appeared to decline over time, but no clear trend could be recognized in the fatal cases as only one time point collection was available (Fig 3A and 3B). Seventeen survived cases showed reduction in viral copy number after 35^th^ POD of which one patient showed persistence of viremia till 76^th^ POD (Fig 3B). Out of 34 cases, urine samples of 5 cases (case numbers-18, 19, 22, 29 and 32) could detect viral RNA copies ranging from 10^4^−10^2^ during initial collection. Semen samples of two survivors (case numbers-32 and 34) (provided consent) were found negative on 32^nd^ and 55^th^ POD.

### Anti-CCHFV IgM and IgG antibody responses in CCHF cases

Serum samples from 124 suspected CCHF cases were screened by ELISA. 27 cases were found positive for anti CCHF IgM ELISA (Table 1). The detection of anti-CCHFV IgM antibodies started from 2^nd^ POD and increased gradually till the last time point of collection i.e 76^th^ POD (Fig 3C). Anti-CCHFV IgG antibodies were absent in the first time point of collection in both survivors and fatal cases. Anti-CCHFV IgG antibodies positivity was observed after 20^th^ POD in two cases; however, most of the survivors were positive by 28^th^ POD (Fig 3D).

The dotted line in Fig 3C and 3D indicates the limit of detection of the assay. The different colour and shapes in Fig 3A, 3B, 3C and 3D indicates individual CCHF survivors. Each case is given a specific colour and shape and the same is followed in Fig 3A, 3B, 3C and 3D.

### Livestock and tick pool samples positivity for CCHFV

A total of 241 Livestock samples (cow, buffalo, goat, sheep, bull, calf) were screened using ELISA and *H*. *annaloticum* tick-pools (n = 162) were screened by real time RT PCR. 21.57% livestock [cow, buffalo, goat, sheep] were found positive for anti-CCHFV IgG antibodies by ELISA while CCHFV RNA were detected in 7.4% tick pools (*H*.*annaloticum*) with copy number ranging from 10^7^−10^4^ (Table 1). The livestock and tick pools positivity denoted the ongoing presence of epizootic cycle of CCHF which has huge zoonotic potential to cause the disease in humans.

### Cytokine profile in serum samples during CCHF infection

**Fig 4:**
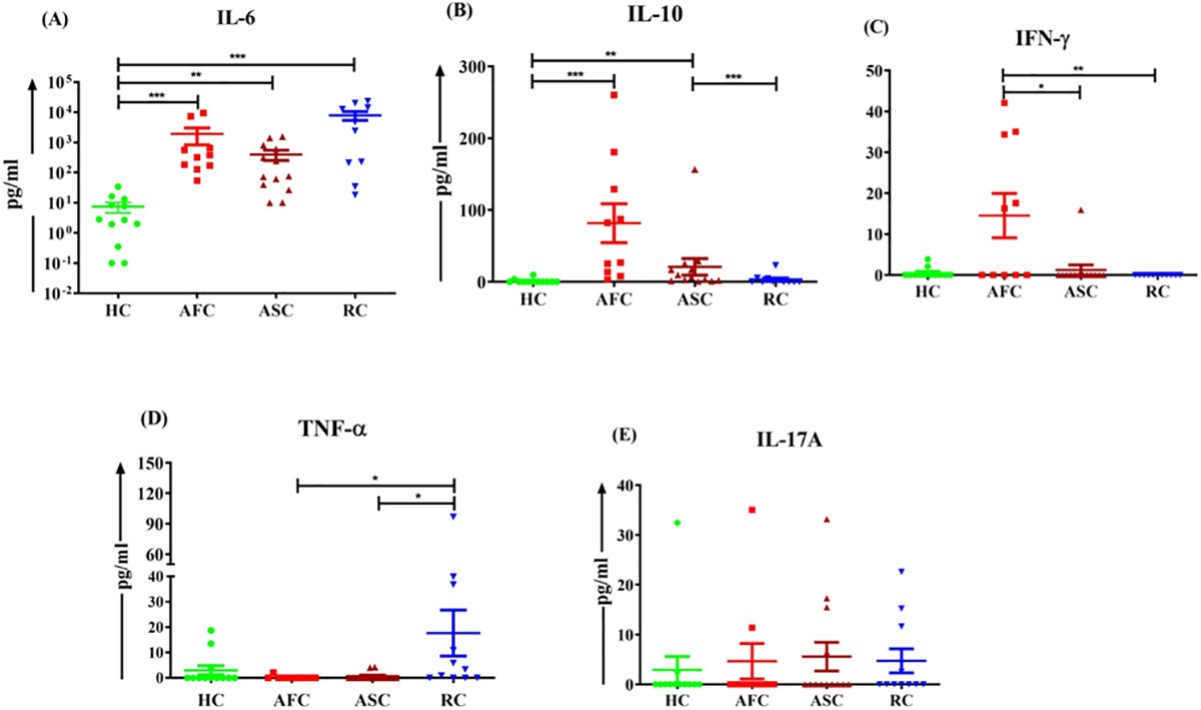
Level of Th1/Th2/Th17 cytokines in the serum samples of acute phase of infection in fatal cases (AFC) (n = 10), acute phase of infection in survived cases (ASC) (n = 13), recovered cases (RC) (n = 11) and healthy controls (HC) (n = 12) were determined using cytometric bead array (CBA) assay. The results are expressed in scatter dot plot of individual values and data are given as Mean ± SEM (pg/ml) of (A) IL-6, (B) IL-10, (C) IFN-γ, (D) TNF-α and (E) IL-17A. Data were analyzed between groups by the non-parametric Kruskal–Wallis test followed by the post-hoc Dunn’s multiple comparison test. The horizontal lines indicate mean values. * P < 0.05; ** P < 0.01; *** P < 0.001.

The serum samples of acute phase of infection in fatal cases (AFC) (n = 10), acute phase of infection in survived cases (ASC) (n = 13), recovered cases (n = 11) and healthy controls (n = 12) were determined using cytometric bead array (CBA) assay for understanding the cytokine profile. The pro-inflammatory cytokine, IL-6 was found significantly higher in the acute phase of fatal cases (Mean ± SE, 1924 pg/ml ± 1091, P<0.001), acute phase of survivors (Mean ± SE, 402.56 pg/ml ± 149.7, P<0.01) and convalescent-phase (Mean ± SE, 7925.88 pg/ml ± 2576, P<0.001) (Fig 4A). The anti-inflammatory cytokine, IL-10 was found significantly elevated in the acute phase of fatal (Mean ± SE, 81.65 pg/ml ± 27.16, P<0.001) and acute phase of survivors (Mean ± SE, 20.83 pg/ml ± 11.61, P<0.01) (Fig 4B). Additionally, the IL-10 level in the acute phase of survived CCHF cases subsided to normal upon recovery (Mean ± SE, 3.21 pg/ml ± 2.05). The level of IFN-γ was found significantly higher in the acute phase of fatal (Mean ± SE, 14.53 pg/ml ± 5.42, P<0.01) and survivors (Mean ± SE, 1.22 pg/ml ± 1.2, P<0.05) (Fig 4C). The level of TNF-α was found significantly increased upon recovery (Mean ± SE, 17.67 pg/ml ± 9.05, P<0.01) (Fig 4D). The serum level of IL-17A was found comparable within the different study groups (Fig 4E). The serum level of IL-2 and IL-4 was below the limit of detection.

### NGS and phylogenetic analysis

Information of the circulating CCHFV strain during the year 2019 is very important to understand if there is introduction of new strains or any reassortments happening in the recent circulating strains. Therefore 29 clinical specimens (27 from EDTA blood and 2 from urine samples) and 5 from tick pools were processed using next generation sequencing and CCHFV sequences were retrieved from these samples. The details of CCHFV genomic sequences which were recovered and reads mapped for the CCHFV sequences are presented in Table 3.

**Table 3:** The percentage of the genome recovered and the reads mapped for the each CCHFV sequences retrieved in this study. The NGS reads were mapped to the L gene (Accession No: KC867272), Asian M gene (Accession No: MN866207), African M gene (Accession No: MH396671), and S gene (Accession No: KX013446).

Sequences	Relevant Read			Total reads	Retrieved Length		
	L	M	S		L	M	S
MCL-19-H-241	1012120	345845	106247	16,05,042.00	12120	5355	1634
MCL-19-H-2006	627206	194335	57755	11,29,464	12120	5349	1634
MCL-19-H-3081	72138	27205	15789	23,24,644	12120	5350	1634
MCL-19-H-1710	1030802	313698	87133	15,18,798	12120	5350	1634
MCL-19-H-1789	1148952	326778	113846	16,26,626	12120	5350	1634
MCL-19-H-387	769186	268389	84651	49,13,742	12120	5348	1634
MCL-19-H-1882	7473	2250	1333	19,25,436	12120	5342	1634
MCL-19-H-3154	105203	48251	15873	16,56,392	11910	5345	1634
MCL-19-H-1574	81692	28353	9869	10,54,358	12120	5346	1634
MCL-19-H-1565	726630	304831	81504	15,60,840	12120	5353	1634
MCL-19-H-2856	395933	102640	68823	28,32,494	12120	5348	1634
MCL-19-H-3092	670183	226030	69377	37,47,382	12120	5342	1634
MCL-19-H-3508	5121	1908	1318	62,01,156	11911	5295	1634
MCL-19-T-2059	83862	53470	15031	12,38,616	12120	5349	1634
MCL-19-H-3110	1356823	688616	483119	88,68,778	12120	5360	1634
MCL-19-H-1812	942096	364576	97360	15,29,536	12118	5358	1634
MCL-19-H-1816	14362	6629	2264	19,34,670	11895	5340	1634
MCL-19-H-99	562158	126505	52823	16,38,634	12120	5359	1634
MCL-19-H-924	23702	6255	3491	11,10,434	12120	5338	1634
MCL-19-H-3075	17574	9113	4192	18,96,776	12120	5339	1634
MCL-19-H-3068	8501	2682	1297	23,95,184	12120	5375	1634
MCL-19-H-2076	2094053	500139	296740	58,36,352	12120	5375	1634
MCL-19-H-2077	23343	5427	4291	13,34,074	12118	5337	1628
MCL-19-T-1989	2823710	990756	288174	45,23,102	12120	5351	1634
MCL-19-H-3232	99334	25164	10774	28,56,082	12119	5346	1634
MCL-19-T-2032	33161	6701	2384	13,37,494	12120	5343	1634
MCL-19-H-1920	5941	2328	799	18,54,722	12120	5341	1626
MCL-19-H-2028	2443	826	515	18,46,258	12045	5330	1600
MCL-19-T-1812	5371	547	1637	8,18,602	11938	1794	1633
MCL-19-H-3460	902702	563948	218017	22,55,190	12141	5361	1635
MCL-19-H-3173	39520	485	5938	16,33,094	12149	5308	1633
MCL-19-H-2525	26206	10001	1950	1,10,008	11967	5321	1604
MCL-19-H-2524	387726	121611	23067	6,82,932	11967	5349	1634

**Fig 5:**
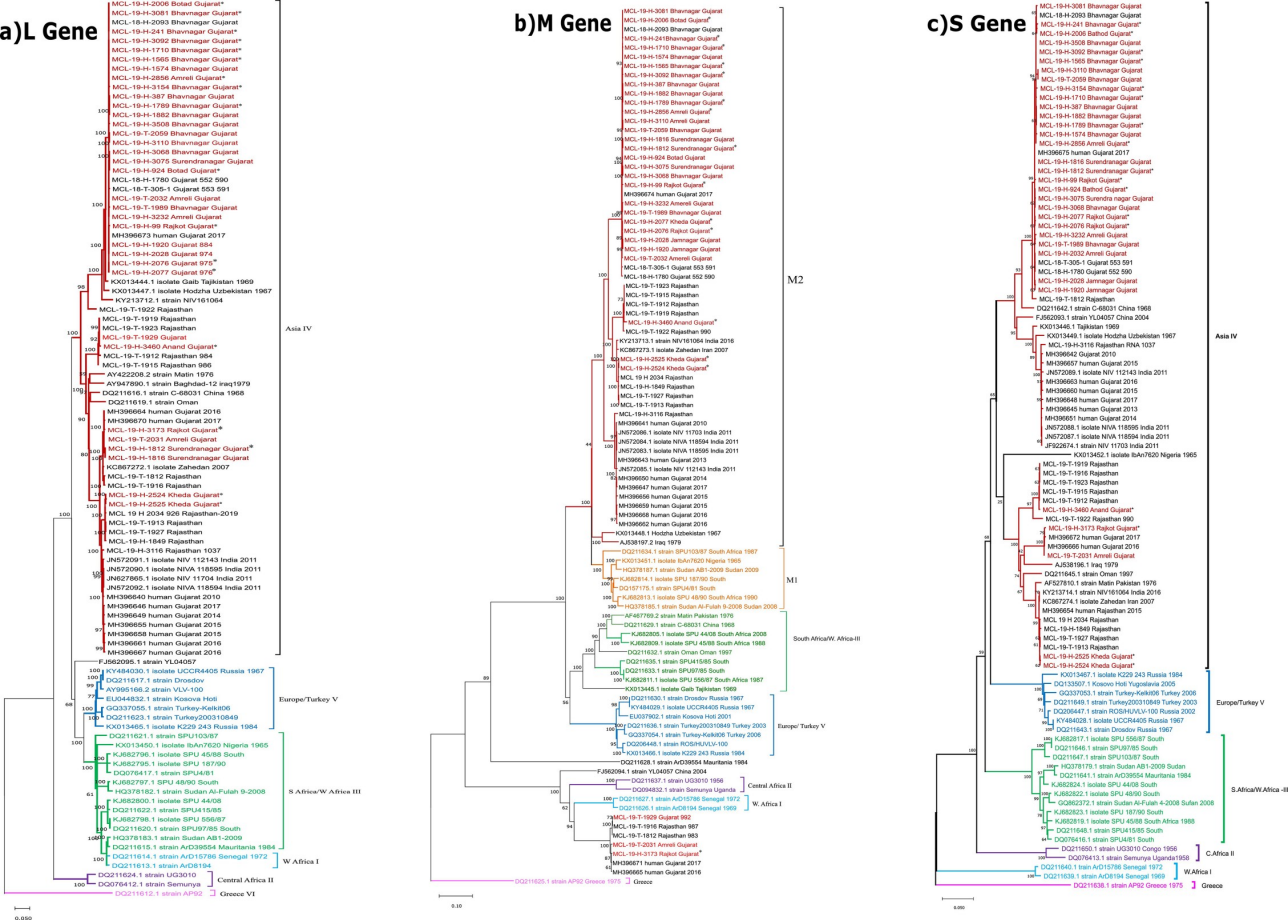
Phylogenetic tree for the L M and S genes for the CCHFV (depicted in A, B and C respectively). Maximum-Likelihood tree for CCHFV sequences retrieved from the clinical samples of the Gujarat state. The tree was constructed using the representative clades downloaded from Genbank using the General-time reversible model with gamma distribution as the rate parameter. The bootstrap replication of 1000 cycles was used to assess the statistical robustness of the generated tree. The scale depicts the number of base substitutions per site. Clades are marked in different colours. * indicates the fatal CCHF cases.

The CCHFV sequences retrieved from the 29 clinical specimens and 5 tick pools were each belonging to L and S gene clustered with the Asian clade. The Asian clade is further grouped into Asia 1 and Asia 2 subgroups [19,20]. The sequences retrieved in this study were found in both the subgroups. Majority of CCHFV sequences from Gujarat clustered with Asia 2 subgroup (Fig 5A and 5B). The remaining small subset of the sequences had proximity with Matin (Pakistan) and Iran sequences that formed a part of Asia 1 subgroup. The percentage nucleotide difference between L gene and the S gene of the Asia 1 and Asia 2 sequences was nearly 9–10% and 8–9% respectively, with respect to the earlier (2010) CCHFV sequence. This indicated the variation within CCHFV nucleotide sequences retrieved from Gujarat, India.

The M gene segment of these 32 CCHFV sequences had a differential clustering. Two sequences, one from tick-pool (MCL-19-T-2031) and one from human (MCL-19-H-3173), clustered with the West Africa-1 clade Senegal sequences, while the remaining 30 CCHFV M gene sequences grouped with the M2 genotype. It was observed that the CCHFV M gene sequences of the West Africa and the two Indian sequences, from this study shared a common ancestor (Fig 5C). The percentage of the nucleotide and amino-acid divergence for M gene was observed to be in the range of 0–8.3% within the M2 genotype, with respect to the earlier (2010) CCHFV sequence. The presence of L and S gene segment in Asian clade and the M gene segment in the African clade is indicative of re-assortment. Apart from the presence of segment in this study earlier CCHFV sequence also clustered to African clade, indicting its earlier presence. The percentage of the nucleotide and amino-acid divergence for each gene is provided in S3 Table and analysis revealed no specific changes associated with the increased mortality.

### CCHF virus isolation

Virus isolation was attempted from the 24 cases with high viral load and twelve CCHFV positive tick-pools. Serum samples from two cases (case numbers-5 and 8) yielded virus isolation on 7^th^ post infection day which was confirmed by qRTPCR with viral RNA copy number of 2.3 x 10^7^ and 1.4 x 10^6^ respectively. None of the tick pools yielded any isolation.

## Discussion

CCHF cases were observed sporadically with increase in the incidence during rainy and winter seasons due to close proximity of livestock and humans, leading to increase possibility of tick bites [19,20]. Occupational vulnerability and risk factors for CCHF have been documented previously amongst the people involved in farming, animal handling, milking the animals, animal grazing, veterinary care providers, slaughtering of animals and nosocomial infection amongst healthcare workers [17,21].

Countries like Turkey had reported highest CCHF cases and an overall mortality rate just under 5% [22]. This study reveals that primary cases (n = 26) with either history of known tick bites or through livestock contacts showed high viral RNA copy number ranging from 10^7^−10^6^ and higher mortality (65.38%). No mortality was observed in secondary cases (n = 8) with viral RNA copy number ranging from 10^6^−10^3^. The high CFR amongst CCHF cases observed in our study implies to the high viral load and bleeding [17,19,20,23,24,25]. Persistence of the CCHF viral RNA without any symptom or sign of active disease in survivors up to 76^th^ POD as noticed in our study has not been reported earlier. This finding of an extended persistence of CCHF viral RNA has a significant implication for public health implying that CCHF cases can remain infective and standard contact precautions needs to be taken during patient care activity. However these facts need to be corroborated further with isolation of CCHFV from the clinical samples to understand the infective potential. Earlier studies by Yagci-Caglayik etal demonstrated detection of CCHFV RNA upto 18 days in serum samples followed by detection until day 14, 17, 16, 18 and 19 in oral swab, nasal swab, urine, faecal swab and sweat swab respectively [26]

We could detect CCHF viral RNA in urine samples in only five cases, suggesting urine is not a good clinical sample for diagnosis. None of the seventeen CCHF survivors whom we had followed showed viral RNA at any time point of collection in the urine samples. The semen samples in two cases after clinical recovery were found to be negative for viral RNA. Further research is needed to understand the infectiousness of the urine and seminal samples.

The findings of leukopenia, thrombocytopenia, elevated PT, INR and aPTT along with SGOT and SGPT in our patients is in concordance with earlier published reports [6,7].

To date, CCHF disease has no definitive proven prophylactic/therapeutic therapy and management options are limited to the administration of ribavirin [27]. Hence, supportive therapy stays as a critical part for case management. Our study also showed clusters of human to human transmission leading to secondary cases which enlightens the importance of contact tracing.

Previous studies indicated an elevated IL-6 levels are consistent with the severity of CCHF [12,24,28] and our study also demonstrated an elevated IL-6 level, indicative of raised pro-inflammatory responses. Besides, increased serum IL-10 level and IFN-γ during the acute phase of the infection are also in line with the previous findings [12,14,29–31]. Interestingly, serum IL-10 level subsided to normal, upon recovery, suggesting its role during the active phase of infection. Additionally, the study indicated increased TNF-α cytokine upon recovery. Although, the present study indicated a mixed pro-inflammatory and anti-inflammatory cytokine response during acute phase, the outcome of the disease could have been dictated by the dominant anti-inflammatory cytokine, IL-10 that lowered to normal upon clearance of the virus in the clinically recovered case.

Findings from the earlier study showed the circulation of different CCHFV strains: S gene of Asia-I and M2 strains [21]. In most of the outbreaks prior to year 2016, intra-genotypic re-assortant of S–Asia-2 and Far East M2 viruses with parental origins in the S (from Tajikistan strain TADJ/HU8966) and L and M (from Afghanistan strain Afg09-2990) was reported [3, 21]. Also, to date from India, the human sequences obtained from previous studies clustered with the Asian lineage [3,4]. In this study, we have identified re-assortment of CCHFV strain from the human sample and tick-pools circulating in Gujarat state in year 2019. The presence of re-assortment of Asian-West African strains within the *Hyalomma* tick pools from Rajasthan state, India and now in human from Gujarat state emphasize the movement of ticks through animal trade and tick bites to human spreading the infection in both the states [5]. We observed that the distribution of the amino acid mutations was not linked to any specific geographical location or increased virulence. However, significant amino-acid mutations were observed in the current reported strains indicating its evolution in India. M-gene West African-Asian strain re-assortment needs further evidence to come to a definitive completion to understand its importation and mixing of two different lineages of CCHFV. The viral RNA presence for a longer time in CCHF infected patients should also be confirmed with virus isolation to understand if they have the transmission capacity.

### Conclusion

This sequential study in CCHF survivors showed for the first time persistence of viral RNA till 76^th^ POD, along with detection of anti-CCHFV IgM antibodies from 2^nd^ POD and while of anti-CCHFV IgG antibodies after 20^th^ POD. This study also identifies the circulation of a re-assortment of Asian-African genotype and Asian genotypes from CCHF cases for the first time from India.

### Limitation of the study

The cell mediated immune responses could not be studied in the fatal and non-fatal cases considering the difficulties in the timely PBMC isolation as samples come from very remote areas of the country. Though efforts for virus isolation was made on all the follow up samples, but the isolation was not successful and the infectiousness for such longer time of viremia could not be established. There are many factors which lead to unsuccessful virus isolation from the clinical samples including the sample quality; cold chain from collection till the laboratory procedure, Ct value of the samples, and above all the chances of successful isolation is itself very low. More sampling and testing of seminal as well as vaginal fluids are required during acute as well as recovery phase to come to the conclusion of sexual transmission of CCHF.

## Supporting information

S1 Table Details of the time points for the follow-up of the CCHF survivors. (DOCX)

S2 Table Laboratory biochemical and haematological parameters of CCHFV cases (n = 34) on the day of admission. (DOCX)

S3 Table The percentage of the nucleotide and amino acid divergence for each gene with respect to the reference CCHFV L, M, S segments having accession number MH396640. (DOCX)
