# Supplementary material for: Sequential determination of viral load, humoral responses and phylogenetic analysis in fatal and non-fatal cases of Crimean-Congo hemorrhagic fever patients from Gujarat, India, 2019
Source: PLoS Negl Trop Dis. 2021 Aug 30;15(8):e0009718. doi: 10.1371/journal.pntd.0009718 (PMC8432894; doi:10.1371/journal.pntd.0009718)
Supplement: S1 Table — (DOCX) [file pntd.0009718.s001.docx]

| **Initial collection at the time of admission (POD range 2-12)** | | **First Follow up (POD range 9-20)** | **Second Follow up (POD range 15-27)** | **Third Follow up (POD range 19-29)** | **Fourth Follow up (POD range 26-35)** | **Fifth Follow up (POD range 32-45)** | **Sixth Follow up (POD range 39-52)** | **Seventh Follow up (POD range 53-59)** | **Eight Follow up (POD range 60-66)** | **Ninth Follow up (POD range 67-72)** | **Tenth Follow up (POD 76)** |
| --- | --- | --- | --- | --- | --- | --- | --- | --- | --- | --- | --- |
| **Survivors cases (n=17)**  **Serial number as per Table-1** | **POD** | **POD** | **POD** | **POD** | **POD** | **POD** | **POD** | **POD** | **POD** | **POD** | **POD** |
| **3** | 6 | 13 | 19 | 25 |  |  |  |  |  |  |  |
| **6** | 6 | 16 | 21 | 25 | 32 |  | 51 |  |  |  |  |
| **10** | 2 | 9 | 12 | 19 | 26 | 32 | 40 |  |  |  |  |
| **12*** | 4 | 12 |  |  |  |  |  |  |  |  |  |
| **14** | 3 |  |  |  |  |  |  |  |  |  |  |
| **15** | 4 | 9 | 15 | 21 |  |  |  |  |  |  |  |
| **16** | 11 | 17 | 24 |  |  |  |  |  |  |  |  |
| **20** | 6 | 14 | 21 | 28 | 35 | 45 | 52 | 59 |  |  |  |
| **22** | 8 | 14 | 22 | 29 | 35 | 43 | 50 |  |  |  |  |
| **23** | 2 | 12 | 20 | 26 |  |  |  |  |  |  |  |
| **24** | 8 | 15 | 23 | 29 |  | 41 |  |  |  |  |  |
| **25** | 8 | 15 | 23 | 29 |  |  |  |  |  |  |  |
| **26** | 9 | 16 |  | 31 |  |  |  |  |  |  |  |
| **28** | 12 |  |  | 28 |  |  |  |  |  |  |  |
| **29** | 4 | 15 | 22 | 29 | 34 | 41 | 49 | 55 | 62 | 69 | 76 |
| **32** | 4 | 12 | 20 | 27 | 35 | 41 | 48 | 55 | 62 |  |  |
| **34** | 3 | 12 | 19 | 25 | 32 | 39 | 46 | 53 | 60 | 67 |  |
| *Case no.12 is real time RTPCR negative and was only anti-CCHFV IgM positive | | | | | | | | | | | |
| The follow up was not possible for case number 14 as patient took discharge against medical advice | | | | | | | | | | | |
| The analysed time point of collection is mentioned as POD (Post Onset Date) which means from date of onset of symptom to the date of collection of sample after admission | | | | | | | | | | | |
| For plotting the graph 3A considering viral RNA log copy number only 16 cases were plotted as one case was real time negative (**different colour and shapes for each cases**) | | | | | | | | | | | |
| For plotting the graph in 3C and 3D, all 17 survivors were considered (**different colour and shapes for each cases**) | | | | | | | | | | | |
| Overlapping of the POD range which are provided in brackets are due to the different time points of follow up of individual cases. | | | | | | | | | | | |

**S1 Table: Details of weekly follow up of CCHF survivors at different POD and time-points**
