# Supplementary material for: Sequential determination of viral load, humoral responses and phylogenetic analysis in fatal and non-fatal cases of Crimean-Congo hemorrhagic fever patients from Gujarat, India, 2019
Source: PLoS Negl Trop Dis. 2021 Aug 30;15(8):e0009718. doi: 10.1371/journal.pntd.0009718 (PMC8432894; doi:10.1371/journal.pntd.0009718)
Supplement: S2 Table — (DOCX) [file pntd.0009718.s002.docx]

**S2 Table: Laboratory biochemical and haematological parameters of CCHFV cases (n=34) on the day of admission**

| Laboratory Parameters (normal values) | CCHF cases (n=34) | | | | | | | | | | | | | | | | | | | | | | | | | | | | | | | | Median values | IQR |
| --- | --- | --- | --- | --- | --- | --- | --- | --- | --- | --- | --- | --- | --- | --- | --- | --- | --- | --- | --- | --- | --- | --- | --- | --- | --- | --- | --- | --- | --- | --- | --- | --- | --- | --- |
|  | 1 | 2 | 3 | 4 | 5 | 6 | 7 | 8 | 9 | 11 | 12 | 14 | 15 | 16 | 17 | 18 | 19 | 20 | 21 | 22 | 23 | 24 | 25 | 26 | 27 | 28 | 29 | 30 | 31 | 32 | 33 | 34 |  |  |
| TLC (4000-10000/µL) | 7300 | 2000 | 3000 | 2400 | 2230 | 1300 | 1600 | 2850 | 3900 | 2200 | 3000 | 1300 | 2200 | 4800 | 3150 | 12000 | 1940 | 7200 | 6900 | 4300 | 4680 | 4500 | 2900 | 2980 | 1720 | 4000 | 15020 | 4000 | 5200 | 4260 | 13200 | 4000 | 3525 | 2487.5 |
| Platelets (150000-450000/ µL) | 91000 | 7000 | 20000 | 25000 | 9000 | 20000 | 8000 | 8000 | 28000 | 33000 | 75000 | 61000 | 132000 | 85000 | 80000 | 7000 | 9000 | 39000 | 24000 | 28000 | 8000 | 60000 | 21000 | 14000 | 10000 | 65000 | 26000 | 20000 | 10000 | 18000 | 15000 | 20000 | 20500 | 34250 |
| AST (24-40 U/L) | 3740 | .. | 144 | 960 | .. | 78 | 3060 | 5413 | .. | 120 | .. | .. | 692.6 | 335 | .. | .. | 21725 | 376 | 655 | 871 | 122 | 86 | 210 | 1904 | .. | .. | .. | 158 | 202 | 288 | 714 | 2071 | 515.5 | 1499 |
| ALT (44-80 U/L) | 12920 | 2433 | 86 | 1110 | 3730 | 48 | 1720 | 1402 | 430 | 49 | .. | 18 | 336.7 | 230 | 156.5 | .. | 5378 | 154 | 610 | 387 | 70 | 48 | 113 | 486 | 116.7 | 515.8 | 81 | 83 | 58 | .. | 180 | 3471 | 230 | 1027 |
| PT (10.30-12.70) | .. | 16.8 | .. | .. | .. | 63 | 12 | 22.2 | 19 | .. | .. | .. | .. | .. | 24.9 | .. | 28 | 16.4 | 23 | .. | 16 | .. | .. | 18.8 | .. | 10.5 | .. | 20 | .. | 23 | 60 | 18 | 19.5 | 6.775 |
| aPTT (30-40 sec) | .. | .. | 60 | 60 | 17 | 50 | 83.9 | 61.1 | 93 | .. | .. | .. | .. | .. | .. | .. | .. | .. | 70 | .. | 50 | .. | .. | 142.6 | .. | 28.7 | 70 | 23 | 81.4 | .. | .. | 70 | 61.1 | 25.7 |
| INR (0.85-1.15) | .. | 1.32 | .. | .. | 1.24 | .. | 1 | .. | 1.59 | 1.69 | .. | .. | .. | .. | 1.95 | .. | .. | 1.39 | .. | .. | .. | .. | .. | .. | .. | 0.95 | .. | 1.53 | 1.25 | .. | 1.2 | .. | 1.32 | 0.34 |
| Serum urea (17-49 mg/dl) | 94 | .. | .. | .. | .. | .. | .. | 100 | 78 | 79.2 | .. | 19.4 | .. | .. | .. | .. | 70 | .. | .. | .. | .. | .. | .. | 38.5 | .. | 49 | .. | .. | 101 | .. | .. | .. | 78 | 45 |

Laboratory parameter for case number 10 was unavailable.

TLC- Total Leucocytes count

AST- serum aspartate aminotransferase

ALT- serum alanine aminotransferase

PT- Prothrombin time

INR- International Normalized Ratio

aPTT- activated partial thromboplastin time

IQR-Inter quartile range
