# Supplementary material for: Sequential determination of viral load, humoral responses and phylogenetic analysis in fatal and non-fatal cases of Crimean-Congo hemorrhagic fever patients from Gujarat, India, 2019
Source: PLoS Negl Trop Dis. 2021 Aug 30;15(8):e0009718. doi: 10.1371/journal.pntd.0009718 (PMC8432894; doi:10.1371/journal.pntd.0009718)
Supplement: S3 Table — (DOCX) [file pntd.0009718.s003.docx]

| **S3 Table: The percentage of the nucleotide and amino acid divergence for each gene with respect to the reference CCHFV L, M, S segments having accession number MH396640** | | | | | | | | |
| --- | --- | --- | --- | --- | --- | --- | --- | --- |
| **L gene** | | | **M gene** | | | **S gene** | | |
| **MH396640_human_Gujarat_2010 (L gene)** | **PND** | **PAD** | **MH396641_human_Gujarat_2010 (M gene)** | **PND** | **PAD** | **MH396642_Gujarat_2010 (S gene)** | **PND** | **PAD** |
| MCL-19-T-2059_Bhavnagar_Gujarat | 11.4 | 2.5 | MCL-19-T-2059_Bhavnagar_Gujarat | 7.5 | 5.4 | MCL-19-T-2059_Bhavnagar_Gujarat | 6.3 | 0.6 |
| MCL-19-T-2032_Amreli_Gujarat | 11.3 | 2.5 | MCL-19-T-2032_Amreli_Gujarat | 7.6 | 5.4 | MCL-19-T-2032_Amreli_Gujarat | 6.0 | 0.4 |
| MCL-19-T-2031_Amreli_Gujarat | 2.8 | 0.8 | MCL-19-T-2031_Amreli_Gujarat | 29.3 | 24.7 | MCL-19-T-2031_Amreli_Gujarat | 10.1 | 1.9 |
| MCL-19-T-1989_Bhavnagar_Gujarat | 11.3 | 2.5 | MCL-19-T-1989_Bhavnagar_Gujarat | 7.6 | 5.4 | MCL-19-T-1989_Bhavnagar_Gujarat | 6.1 | 0.4 |
| MCL-19-T-1927_Rajasthan | 2.1 | 0.8 | MCL-19-T-1927_Rajasthan | 6.9 | 5.6 | MCL-19-T-1927_Rajasthan | 9.3 | 2.3 |
| MCL-19-T-1923_Rajasthan | 8.1 | 1.9 | MCL-19-T-1923_Rajasthan | 8.3 | 6.4 | MCL-19-T-1923_Rajasthan | 9.5 | 1.9 |
| MCL-19-T-1922_Rajasthan | 9.3 | 2.2 | MCL-19-T-1922_Rajasthan | 8.2 | 6.4 | MCL-19-T-1922_Rajasthan | 8.7 | 1.2 |
| MCL-19-T-1919_Rajasthan | 8.0 | 1.9 | MCL-19-T-1919_Rajasthan | 8.2 | 6.4 | MCL-19-T-1919_Rajasthan | 9.5 | 1.9 |
| MCL-19-T-1916_Rajasthan | 2.6 | 0.8 | MCL-19-T-1916_Rajasthan | 29.4 | 24.5 | MCL-19-T-1916_Rajasthan | 9.5 | 1.9 |
| MCL-19-T-1915_Rajasthan | 8.1 | 2.0 | MCL-19-T-1915_Rajasthan | 8.3 | 6.5 | MCL-19-T-1915_Rajasthan | 9.6 | 1.9 |
| MCL-19-T-1913_Rajasthan | 2.1 | 0.8 | MCL-19-T-1913_Rajasthan | 6.9 | 5.6 | MCL-19-T-1913_Rajasthan | 9.3 | 2.3 |
| MCL-19-T-1912_Rajasthan | 8.2 | 2.1 | MCL-19-T-1912_Rajasthan | 8.2 | 6.4 | MCL-19-T-1912_Rajasthan | 9.6 | 1.9 |
| MCL-19-T-1812_Rajasthan | 2.6 | 0.7 | MCL-19-T-1812_Rajasthan | 29.4 | 24.6 | MCL-19-T-1812_Rajasthan | 5.8 | 0.4 |
| MCL-19-H-99_Rajkot_Gujarat | 11.3 | 2.5 | MCL-19-H-99_Rajkot_Gujarat | 7.6 | 5.2 | MCL-19-H-99_Rajkot_Gujarat | 6.0 | 0.4 |
| MCL-19-H-924_Batod_Gujarat | 11.4 | 2.5 | MCL-19-H-924_Botad_Gujarat | 7.6 | 5.3 | MCL-19-H-924_Bathod_Gujarat | 5.9 | 0.4 |
| MCL-19-H-387_Bhavnagar_Gujarat | 11.4 | 2.6 | MCL-19-H-387_Bhavnagar_Gujarat | 7.6 | 5.3 | MCL-19-H-387_Bhavnagar_Gujarat | 6.1 | 0.4 |
| MCL-19-H-3508_Bhavnagar_Gujarat | 11.4 | 2.5 | MCL-19-H-3508_Bhavnagar_Gujarat | 12.8 | 12.8 | MCL-19-H-3508_Bhavnagar_Gujarat | 6.1 | 0.4 |
| MCL-19-H-3460_Anand_Gujarat | 8.1 | 2.0 | MCL-19-H-3460_Anand_Gujarat | 8.2 | 6.4 | MCL-19-H-3460_Anand_Gujarat | 9.5 | 1.9 |
| MCL-19-H-3232_Amreli_Gujarat | 11.3 | 2.4 | MCL-19-H-3232_Amreli_Gujarat | 7.6 | 5.3 | MCL-19-H-3232_Amreli_Gujarat | 6.0 | 0.4 |
| MCL-19-H-3173_Rajkot_Gujarat | 2.7 | 0.8 | MCL-19-H-3173_Rajkot_Gujarat | 29.2 | 24.6 | MCL-19-H-3173_Rajkot_Gujarat | 10.4 | 1.9 |
| MCL-19-H-3154_Bhavnagar_Gujarat | 11.3 | 2.5 | MCL-19-H-3154_Bhavnagar_Gujarat | 7.5 | 5.2 | MCL-19-H-3154_Bhavnagar_Gujarat | 6.2 | 0.6 |
| MCL-19-H-3116_Rajasthan | 1.0 | 0.3 | MCL-19-H-3116_Rajasthan | 1.4 | 1.7 | MCL-19-H-3116_Rajasthan | 0.8 | 0.6 |
| MCL-19-H-3110_Bhavnagar_Gujarat | 11.4 | 2.5 | MCL-19-H-3110_Bhavnagar_Gujarat | 7.5 | 5.3 | MCL-19-H-3110_Bhavnagar_Gujarat | 6.3 | 0.6 |
| MCL-19-H-3092_Bhavnagar_Gujarat | 11.3 | 2.5 | MCL-19-H-3092_Bhavnagar_Gujarat | 7.6 | 5.5 | MCL-19-H-3092_Bhavnagar_Gujarat | 6.1 | 0.4 |
| MCL-19-H-3081_Bhavnagar_Gujarat | 11.3 | 2.5 | MCL-19-H-3081_Bhavnagar_Gujarat | 7.6 | 5.5 | MCL-19-H-3081_Bhavnagar_Gujarat | 6.2 | 0.4 |
| MCL-19-H-3075_Surendra_Nagar_Gujarat | 11.4 | 2.6 | MCL-19-H-3075_Surendra_Nagar_Gujarat | 7.6 | 5.2 | MCL-19-H-3075_Surendra_nagar_Gujarat | 5.9 | 0.4 |
| MCL-19-H-3068_Bhavnagar_Gujarat | 11.4 | 2.6 | MCL-19-H-3068_Bhavnagar_Gujarat | 7.7 | 5.2 | MCL-19-H-3068_Bhavnagar_Gujarat | 5.9 | 0.4 |
| MCL-19-H-2856_Amreli_Gujarat | 11.3 | 2.5 | MCL-19-H-2856_Amreli_Gujarat | 7.6 | 5.3 | MCL-19-H-2856_Amreli_Gujarat | 6.1 | 0.4 |
| MCL-19-H-2525_Kheda_Gujarat | 2.1 | 0.9 | MCL-19-H-2525_Kheda_Gujarat | 6.8 | 5.5 | MCL-19-H-2525_Rajkot_Gujarat | 9.3 | 2.3 |
| MCL-19-H-2524_Kheda_Gujarat | 2.1 | 0.9 | MCL-19-H-2524_Kheda_Gujarat | 6.8 | 5.5 | MCL-19-H-2524_Kheda_Gujarat | 9.3 | 2.3 |
| MCL-19-H-241_Bhavnagar_Gujarat | 11.3 | 2.5 | MCL-19-H-241_Bhavnagar_Gujarat | 7.5 | 5.4 | MCL-19-H-241_Bhavnagar_Gujarat | 6.2 | 0.2 |
| MCL-19-H-2077_Rajkot_Gujarat | 11.3 | 2.5 | MCL-19-H-2077_Rajkot_Gujarat | 7.5 | 5.3 | MCL-19-H-2077_Rajkot_Gujarat | 6.0 | 0.4 |
| MCL-19-H-2076_Rajkot_Gujarat | 11.3 | 2.5 | MCL-19-H-2076_Rajkot_Gujarat | 7.5 | 5.3 | MCL-19-H-2076_Rajkot_Gujarat | 6.0 | 0.4 |
| MCL-19-H-2028_Jamnagar_Gujarat | 11.4 | 2.5 | MCL-19-H-2028_Jamnagar_Gujarat | 7.5 | 5.4 | MCL-19-H-2028_Jamnagar_Gujarat | 6.0 | 0.4 |
| MCL-19-H-2006_Botad_Gujarat | 11.3 | 2.4 | MCL-19-H-2006_Botad_Gujarat | 7.6 | 5.4 | MCL-19-H-2006_Bathod_Gujarat | 6.2 | 0.4 |
| MCL-19-H-1920_Jamnagar_Gujarat | 11.3 | 2.5 | MCL-19-H-1920_Jamnagar_Gujarat | 7.5 | 5.4 | MCL-19-H-1920_Jamnagar_Gujarat | 6.0 | 0.4 |
| MCL-19-H-1882_Bhavnagar_Gujarat | 11.4 | 2.6 | MCL-19-H-1882_Bhavnagar_Gujarat | 7.7 | 5.4 | MCL-19-H-1882_Bhavnagar_Gujarat | 6.1 | 0.4 |
| MCL-19-H-1849_Rajasthan | 2.0 | 0.8 | MCL-19-H-1849_Rajasthan | 6.9 | 5.8 | MCL-19-H-1849_Rajasthan | 9.3 | 2.3 |
| MCL-19-H-1816_Surendranagar_Gujarat | 2.7 | 0.8 | MCL-19-H-1816_Surendranagar_Gujarat | 7.7 | 5.3 | MCL-19-H-1816_Surendranagar_Gujarat | 5.9 | 0.4 |
| MCL-19-H-1812_Surendranagar_Gujarat | 2.6 | 0.8 | MCL-19-H-1812_Surendranagar_Gujarat | 7.7 | 5.3 | MCL-19-H-1812_Surendranagar_Gujarat | 5.9 | 0.4 |
| MCL-19-H-1789_Bhavnagar_Gujarat | 11.4 | 2.5 | MCL-19-H-1789_Bhavnagar_Gujarat | 7.7 | 5.4 | MCL-19-H-1789_Bhavnagar_Gujarat | 6.1 | 0.4 |
| MCL-19-H-1710_Bhavnagar_Gujarat | 11.4 | 2.5 | MCL-19-H-1710_Bhavnagar_Gujarat | 7.6 | 5.4 | MCL-19-H-1710_Bhavnagar_Gujarat | 6.1 | 0.4 |
| MCL-19-H-1574_Bhavnagar_Gujarat | 11.4 | 2.5 | MCL-19-H-1574_Bhavnagar_Gujarat | 7.6 | 5.3 | MCL-19-H-1574_Bhavnagar_Gujarat | 6.1 | 0.4 |
| MCL-19-H-1565_Bhavnagar_Gujarat | 11.3 | 2.4 | MCL-19-H-1565_Bhavnagar_Gujarat | 7.6 | 5.3 | MCL-19-H-1565_Bhavnagar_Gujarat | 6.1 | 0.4 |
| MCL_19_H_2034_Rajasthan | 2.1 | 0.8 | MCL_19_H_2034_Rajasthan | 6.8 | 5.6 | MCL_19_H_2034_Rajasthan | 9.2 | 2.3 |
| KY484030.1_isolate_UCCR4405_Russia_1967 | 12.4 | 3.0 | KY484029.1_isolate_UCCR4405_Russia_1967 | 19.2 | 14.9 | KY484028.1_isolate_UCCR4405_Russia_1967 | 11.3 | 3.5 |
| KY213712.1_strain_NIV161064 | 11.0 | 2.4 | KY213713.1_strain_NIV161064_India_2016 | 6.8 | 5.5 | KY213714.1_strain_NIV161064_India_2016 | 9.3 | 2.3 |
| KX013465.1_isolate_K229_243_Russia_1984 | 12.4 | 3.1 | KX013466.1_isolate_K229_243_Russia_1984 | 19.0 | 14.9 | KX013467.1_isolate_K229_243_Russia_1984 | 11.7 | 4.4 |
| KX013450.1_isolate_IbAn7620_Nigeria_1965 | 13.7 | 4.1 | KX013451.1_isolate_IbAn7620_Nigeria_1965 | 11.9 | 8.6 | KX013452.1_isolate_IbAn7620_Nigeria_1965 | 11.5 | 3.7 |
| KX013447.1_isolate_Hodzha_Uzbekistan_1967 | 11.2 | 2.6 | KX013448.1_Hodzha_Uzbekistan_1967 | 10.7 | 8.0 | KX013449.1_isolate_Hodzha_Uzbekistan_1967 | 2.1 | 1.9 |
| KX013444.1_isolate_Gaib_Tajikistan_1969 | 11.2 | 2.5 | KX013445.1_isolate_Gaib_Tajikistan_1969 | 18.9 | 12.9 | KX013446.1_Tajikistan_1969 | 2.0 | 0.4 |
| KJ682800.1_isolate_SPU_44/08 | 13.1 | 3.2 | KJ682814.1_isolate_SPU_187/90_South | 11.5 | 7.8 | KJ682824.1_isolate_SPU_44/08_South | 11.9 | 2.5 |
| KJ682798.1_isolate_SPU_556/87 | 13.1 | 3.2 | KJ682813.1_isolate_SPU_48/90_South_Africa_1990 | 12.1 | 8.7 | KJ682823.1_isolate_SPU_187/90_South | 12.3 | 2.5 |
| KJ682797.1_SPU_48/90_South | 13.1 | 3.3 | KJ682811.1_isolate_SPU_556/87_South_Africa_1987 | 18.4 | 13.8 | KJ682822.1_isolate_SPU_48/90_South | 12.5 | 2.7 |
| KJ682796.1_isolate_SPU_45/88_South | 13.1 | 3.3 | KJ682809.1_isolate_SPU_45/88_South_Africa_1988 | 19.1 | 14.3 | KJ682819.1_isolate_SPU_45/88_South_Africa_1988 | 12.7 | 2.3 |
| KJ682795.1_isolate_SPU_187/90 | 13.0 | 3.2 | KJ682805.1_isolate_SPU_44/08_South_Africa_2008 | 19.5 | 14.7 | KJ682817.1_isolate_SPU_556/87_South | 12.0 | 2.1 |
| KC867272.1_isolate_Zahedan_2007 | 2.4 | 0.6 | KC867273.1_isolate_Zahedan_Iran_2007 | 6.9 | 6.0 | KC867274.1_isolate_Zahedan_Iran_2007 | 9.3 | 2.3 |
| JN627865.1_isolate_NIV_11704_India_2011 | 0.3 | 0.2 | JN572086.1_isolate_NIV_11703_India_2011 | 0.2 | 0.2 | JN572089.1_isolate_NIV_112143_India_2011 | 0.4 | 0.4 |
| JN572092.1_isolate_NIVA_118594_India_2011 | 0.3 | 0.2 | JN572085.1_isolate_NIV_112143_India_2011 | 0.3 | 0.2 | JN572088.1_isolate_NIVA_118595_India_2011 | 0.2 | 0.2 |
| JN572091.1_isolate_NIV_112143_India_2011 | 0.4 | 0.1 | JN572084.1_isolate_NIVA_118594_India_2011 | 0.3 | 0.2 | JN572087.1_isolate_NIVA_118594_India_2011 | 0.2 | 0.2 |
| JN572090.1_isolate_NIVA_118595_India_2011 | 0.4 | 0.3 | JN572083.1_isolate_NIVA_118595_India_2011 | 0.2 | 0.2 | JF922674.1_strain_NIV_11703_India_2011 | 0.2 | 0.2 |
| HQ378183.1_strain_Sudan_AB1-2009 | 13.2 | 3.2 | HQ378187.1_strain_Sudan_AB1-2009_Sudan_2009 | 11.9 | 8.1 | HQ378179.1_strain_Sudan_AB1-2009_Sudan | 13.2 | 2.3 |
| HQ378182.1_strain_Sudan_Al-Fulah_9-2008 | 13.3 | 3.2 | HQ378185.1_strain_Sudan_Al-Fulah_9-2008_Sudan_2008 | 12.4 | 8.5 | GQ862372.1_strain_Sudan_Al-Fulah_4-2008_Sufan_2008 | 12.6 | 2.5 |
| GQ337055.1_strain_Turkey-Kelkit06 | 12.6 | 2.9 | GQ337054.1_strain_Turkey-Kelkit06_Turkey_2006 | 19.1 | 15.0 | GQ337053.1_strain_Turkey-Kelkit06_Turkey_2006 | 11.6 | 4.6 |
| FJ562095.1_strain_YL04057 | 12.0 | 2.7 | FJ562094.1_strain_YL04057_China_2004 | 28.5 | 25.0 | FJ562093.1_strain_YL04057_China_2004 | 5.7 | 1.5 |
| EU044832.1_strain_Kosova_Hoti | 12.5 | 3.0 | EU037902.1_strain_Kosova_Hoti_2001 | 19.3 | 15.0 | DQ133507.1_Kosovo_Hoti_Yugoslavia_2005 | 10.6 | 3.3 |
| DQ211624.1_strain_UG3010 | 15.6 | 4.6 | DQ211637.1_strain_UG3010_1956 | 29.6 | 25.7 | DQ211650.1_strain_UG3010_Congo_1956 | 12.2 | 3.1 |
| DQ211623.1_strain_Turkey200310849 | 12.6 | 2.8 | DQ211636.1_strain_Turkey200310849_Turkey_2003 | 18.8 | 14.7 | DQ211649.1_strain_Turkey200310849_Turkey_2003 | 11.2 | 3.3 |
| DQ211622.1_strain_SPU415/85 | 13.1 | 3.2 | DQ211635.1_strain_SPU415/85_South | 18.6 | 13.8 | DQ211648.1_strain_SPU415/85_South | 12.9 | 2.9 |
| DQ211621.1_strain_SPU103/87 | 12.6 | 3.2 | DQ211634.1_strain_SPU103/87_South_Africa_1987 | 11.8 | 8.7 | DQ211647.1_strain_SPU103/87_South | 12.0 | 2.1 |
| DQ211620.1_strain_SPU97/85_South | 13.1 | 3.2 | DQ211633.1_strain_SPU97/85_South | 18.3 | 13.7 | DQ211646.1_strain_SPU97/85_South | 12.0 | 2.1 |
| DQ211619.1_strain_Oman | 7.9 | 1.6 | DQ211632.1_strain_Oman_Oman_1997 | 18.8 | 12.9 | DQ211645.1_strain_Oman_1997 | 9.2 | 2.1 |
| DQ211617.1_strain_Drosdov | 12.4 | 3.0 | DQ211630.1_strain_Drosdov_Russia_1967 | 19.2 | 14.9 | DQ211643.1_strain_Drosdov_Russia_1967 | 11.3 | 3.5 |
| DQ211616.1_strain_C-68031_China_1968 | 6.7 | 1.5 | DQ211629.1_strain_C-68031_China_1968 | 18.5 | 13.2 | DQ211642.1_strain_C-68031_China_1968 | 6.1 | 0.8 |
| DQ211615.1_strain_ArD39554_Mauritania_1984 | 13.2 | 3.3 | DQ211628.1_strain_ArD39554_Mauritania_1984 | 28.4 | 23.1 | DQ211641.1_strain_ArD39554_Mauritania_1984 | 12.8 | 2.3 |
| DQ211614.1_strain_ArD15786_Senegal_1972 | 13.3 | 3.5 | DQ211627.1_strain_ArD15786_Senegal_1972 | 29.3 | 25.4 | DQ211640.1_train_ArD15786_Senegal_1972 | 16.7 | 5.0 |
| DQ211613.1_strain_ArD8194 | 13.2 | 3.3 | DQ211626.1_strain_ArD8194_Senegal_1969 | 29.1 | 24.9 | DQ211639.1_strain_ArD8194_Senegal_1969 | 16.5 | 5.6 |
| DQ211612.1_strain_AP92 | 21.2 | 8.6 | DQ211625.1_strain_AP92_Greece_1975 | 28.2 | 25.4 | DQ211638.1_strain_AP92_Greece_1975 | 17.0 | 7.9 |
| AY995166.2_strain_VLV-100 | 12.4 | 3.0 | DQ206448.1_strain_ROS/HUVLV-100 | 18.9 | 14.7 | DQ206447.1_strain_ROS/HUVLV-100_Russia_2002 | 11.1 | 3.5 |
| DQ076417.1_strain_SPU4/81 | 13.2 | 3.4 | DQ157175.1_strain_SPU4/81_South | 11.7 | 8.3 | DQ076416.1_strain_SPU4/81_South | 12.2 | 2.3 |
| DQ076412.1_strain_Semunya | 15.6 | 4.3 | DQ094832.1_strain_Semunya_Uganda | 29.8 | 26.0 | DQ076413.1_strain_Semunya_Uganda1958 | 12.9 | 2.9 |
| AY947890.1_strain_Baghdad-12_iraq1979 | 7.7 | 2.1 | AJ538197.2_Iraq_1979 | 11.7 | 8.1 | AJ538196.1_Iraq_1979 | 9.9 | 3.3 |
| AY422208.2_strain_Matin_1976 | 7.8 | 1.7 | AF467769.2_strain_Matin_Pakistan_1976 | 18.5 | 13.4 | AF527810.1_strain_Matin_Pakistan_1976 | 9.5 | 2.1 |
